# Supplementary material for: Inhibition Mechanism of Lactiplantibacillus plantarum on the Growth and Biogenic Amine Production in Morganella morganii
Source: Foods. 2023 Sep 29;12(19):3625. doi: 10.3390/foods12193625 (PMC10572400; doi:10.3390/foods12193625)
Supplement: Supplementary file 1 [file foods-12-03625-s001.zip › foods-2619023-supplementary.pdf]

foods

Table S1. Effect of different condition treatments on the biogenic amine production capacity of *Morganella morganii.* [a]

Biogenic Amine Content (mg/L)	Control Group [b]				Experimental Group [b]			
	pH				pH			
	4	5	6	7	4	5	6	7
Phenylethylamine	0.32 ± 0.01 [f]	10.63 ± 2.01 [e]	13.40 ± 2.69 [e]	18.41 ± 4.68 [d]	0.40 ± 0.05 [f]	0.37 ± 0.06 [f]	10.34 ± 1.55 [e]	14.49 ± 1.06 [d,e]
Tryptamine	0.16 ± 0.02 [d]	0.16 ± 0.01 [d]	0.17 ± 0.02 [d]	ND [c,e]	0.21 ± 0.04 [d]	0.21 ± 0.03 [d]	0.18 ± 0.05 [d]	0.16 ± 0.02 [d]
Cadaverine	2.94 ± 0.02 [g]	15.91 ± 2.07 [e]	33.64 ± 6.30 [d]	18.87 ± 1.67 [e]	3.25 ± 0.18 [f,g]	2.68 ± 0.22 [g]	8.54 ± 1.43 [f]	17.52 ± 2.81 [e]
Putrescine	0.63 ± 0.01 [h]	7.69 ± 0.61 [f,g]	11.01 ± 2.11 [e]	31.99 ± 3.26 [d]	0.65 ± 0.02 [h]	1.04 ± 0.02 [h]	5.84 ± 1.31 [g]	9.98 ± 1.85 [e,f]
Tyramine	2.04 ± 0.09 [d,e]	2.32 ± 0.34 [d]	2.45 ± 0.46 [d]	2.23 ± 0.27 [d]	2.13 ± 0.29 [de]	1.95 ± 0.21 [d,e,f]	1.38 ± 0.22 [f]	1.61 ± 0.31 [e,f]
Histamine	0.4 ± 0.01 [h]	33.04 ± 2.53 [d,e]	36.94 ± 4.91 [d]	21.40 ± 3.20 [g]	0.7 ± 0.03 [h]	0.63 ± 0.03 [h]	27.74 ± 2.59 [e,f]	25.17 ± 4.12 [f,g]

[a] Values are expressed as mean ± standard deviation (n = 3). [b] *M. morganii* cultured in blank medium is the control group; *M. morganii* cultured in medium supplemented with *Lpb. plantarum* CFS is the experimental group. [c] ND, the biogenic amine of the samples is less than 0.05 mg/L in wet base content. [d–h] Different letters in the same row indicate a significant difference ($P < 0.05$, different culture conditions).
